# Cancer Immunotherapy and Immune-Related Adverse Events: Focusing on Non-Small Cell Lung Cancer

**DOI:** 10.3390/curroncol33100607

**Published:** 2026-10-09

**Authors:** Atif Kamil, Sara Cowles, Casaundra C. Driver, Aakriti Arora, Madison M. Swyers, Luke W. Boldt, Mahvish Muzaffar, Sameer A. Batoo, Li V. Yang

**Affiliations:** Department of Internal Medicine, Brody School of Medicine, East Carolina University, Greenville, NC 27834, USA; kamila23@ecu.edu (A.K.); driverc21@ecualumni.ecu.edu (C.C.D.); swyersm23@students.ecu.edu (M.M.S.); lucas_boldt@med.unc.edu (L.W.B.); muzaffarm@ecu.edu (M.M.)

**Keywords:** cancer immunotherapy, immune checkpoint inhibitors, immune-related adverse events (irAEs), inflammation, non-small cell lung cancer (NSCLC)

## Abstract

The development of immunotherapy has changed the paradigm of cancer treatment. In particular, immune checkpoint inhibitors (ICIs) demonstrate therapeutic efficacy and improve patient survival across multiple cancer types. Currently, however, only a subset of cancer patients can achieve objective therapeutic responses and gain survival benefits from immunotherapy. Treatment-associated side effects, namely immune-related adverse events (irAEs), may cause autoimmune and inflammatory complications in various organ systems and hinder the use of immunotherapy in cancer patients. Herein, the development of cancer immunotherapy and the modulatory roles of the tumor immune microenvironment, with a focus on ICI therapy in non-small cell lung cancer (NSCLC), are reviewed. The grading, types, biomarkers, and treatment of irAEs are also discussed. More research is needed to further increase the efficacy of cancer immunotherapy and alleviate irAEs.

## Simple Summary

Immunotherapy harnesses the power of the patient’s own immune system to target and destroy cancer cells. As an important type of immunotherapy, immune checkpoint inhibitors have changed the landscape of cancer therapy and significantly improved patient outcomes. However, only some patients respond to immunotherapy and gain benefits from the treatment. Immunotherapy may also cause inflammatory and autoimmune side effects, known as immune-related adverse events. This review discusses the development of cancer immunotherapy, the modulatory effects of tumor microenvironments on immunotherapy, the use of immune checkpoint inhibitors in non-small cell lung cancer, and the types, management, and biomarkers of immunotherapy-related side effects.

## 1. Overview of Cancer Immunotherapy

**Figure 1:**
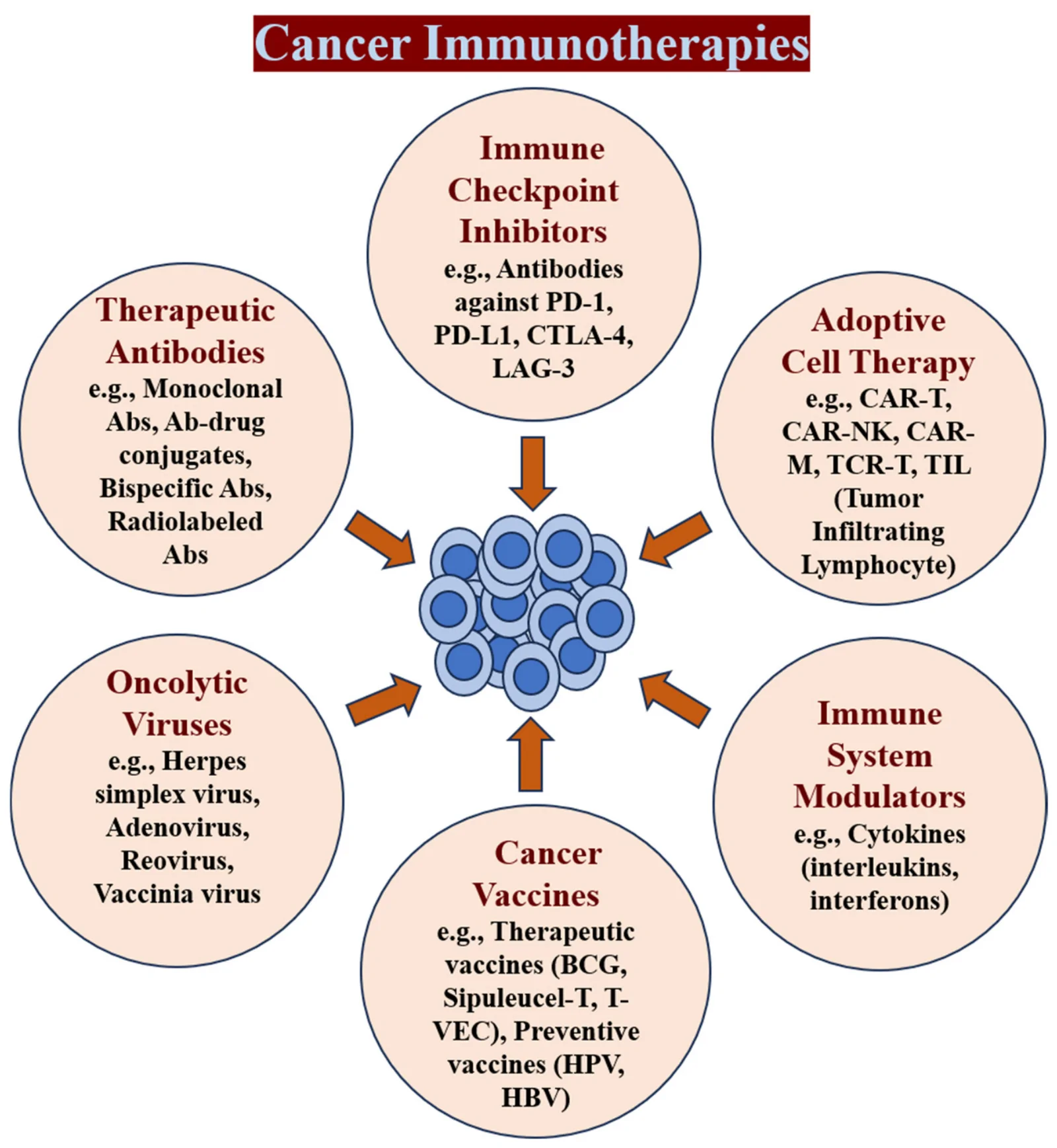
Approaches and modalities used for cancer immunotherapy.

Cancer is caused by the accumulation of gene mutations [1,2], which can generate tumor neoantigens. These neoantigens are recognized by the immune system as foreign molecules and elicit immune responses [3]. Immune cells infiltrate the tumor microenvironment, contributing significantly to changes in tumor progression and immunosurveillance [4]. The immune system has evolved several complex mechanisms to detect and kill cancer cells to protect the body from malignancy. However, cancer cells are also capable of evading immunosurveillance. Cancer immunoediting protects tumor growth and shapes the tumor’s immune responses in a three-stage process, i.e., elimination, equilibrium, and escape. Whereas the innate and adaptive immune systems recognize and attempt to eliminate tumor antigens, some cancer cells evade and reach an equilibrium state. When selective tumor cells develop resistance to the anti-cancer immune responses, they escape through different mechanisms and pathways, such as alteration of cytokine expression, modification/loss of antigens, and modulation of immune checkpoint proteins [5]. The standard treatment procedures for cancer are surgery, radiation, and chemotherapy. However, cancer immunotherapy tailored to the patient’s immunological context is emerging as a promising approach for activating the immune system to exert antitumor effects (Figure 1) [2].

### 1.1. Brief History of Cancer Immunotherapy

The concept of immune response for the eradication of bacterial and viral infections is at least a century old; however, the accumulation of leukocytes in neoplastic tissues, resulting in an association between inflammation and tumor, was reported in 1863 [6]. The early cancer immunotherapy attempt was to treat sarcoma patients with a combination of live and inactivated *Streptococcus pyogenes* and *Serratia marcescens*. This pioneering work provided proof of concept for immunotherapy, but unknown mechanisms, variable results, and infection risks deterred the progress [7,8]. Thereafter, oncolytic viruses (genetically altered viruses), having the capability to specifically infect tumor cells and generate a cascade of antitumor immune responses, were introduced. The recent progress in the use of oncolytic viruses could be attributed to the development of genetic modification strategies [9].

Anti-cancer vaccines were introduced for cancer immunotherapy and were designed to activate antitumor T cell responses against cancer antigens, e.g., melanoma-derived antigens (MZ2-D and MZ2-E). The human melanoma antigen proved highly potent not only in in vivo tumor rejection, but also in the immune response of infiltrating lymphocytes into the tumor tissue [10,11]. Dendritic cells (DCs) are professional antigen-presenting cells (APCs); upon activation, they internalize, process and present epitopes to T cells for the induction of cytotoxic T cell responses [12]. Thereby, DC-based anti-cancer vaccines showed promising results, such as sipuleucel-T in the treatment of advanced prostate cancer [13]. Whole tumor cell-based vaccines are also promising in many cancer types [14].

Immune cells release cytokines for quick and organized dissemination of immune signals to stimulate cell interaction, communication, and response to immune stress such as infection, inflammation, and tumors [15]. Interleukin 2 (IL-2) injection in large quantities could restrict metastatic cancer [16,17]. Similarly, a large family of cytokines called interferons are cancer therapeutic agents. Interferon-alpha (IFN-α) plays crucial roles in eradicating cancer by inducing senescence and apoptosis. IFN-α also activates a cascade of anti-cancer immune responses for the induction of DC and T-cell cytotoxicity on tumor cells [18]. IFN-α has less tolerance and high toxicity but is still considered a useful therapeutic in chronic myeloid leukemia and melanoma in high dosages. Cytokines are considered for treating cancer in combination with other immunotherapies.

Adoptive cell therapy (ACT) is based on patient-derived or engineered T cells, which are expanded ex vivo and then reinfused back into patients to kill tumor cells [19]. ACT in combination with IL-2 is more effective in metastatic cancers [17]. The clonal expansion of specific T cells against highly expressed and mutated cancer antigens was targeted for ACT in patients with metastatic melanoma. Engineered T cells against tumor antigens or neoantigens were applied in adoptive transfer technology [20].

The chimeric antigen receptor (CAR)-T cell therapy is an emerging immunotherapy for the highly specific and rapid eradication of cancer cells. CAR-T cell therapy has shown a significant improvement in cancer-related symptoms and prolonged cancer immunity. At present, two types of engineered T cells, including CAR-T cells and T-cell receptor (TCR)-engineered T cells, have gained popularity in the fight against malignant tumors. CAR-T-cell therapies are based on the complementarity of the antibody fragments for the recognition of specific tumor antigens. The CAR protein molecules are engineered to enable a highly specific anti-cancer ability of immune cells, such as T cells, natural killer (NK) cells, and macrophages. CAR-T, CAR-NK, and CAR-M are genetically altered cells produced by transducing them with a CAR. CAR molecules are membrane-anchored cell-surface proteins that comprise an antigen-binding domain, a hinge region, a transmembrane domain, one or more costimulatory domains, and an activation domain [21]. The antigen-binding domain is an extracellular single-chain variable fragment (scFv), sometimes coupled with other peptides/proteins. The hinge and transmembrane regions are made of the IgG4 hinge and the CD28 transmembrane domain, which helps connect the extracellular and intracellular domains of the CAR molecule. CD28 and CD137 serve as costimulatory domains for enhancing cytokine secretion, T cell proliferation, and antitumor activity. CD3ζ serves as an activation domain for transducing extracellular signals to activate T cells for cytolysis and cytokine release [22]. Four different versions of CAR-T cells have been developed for the improved killing of cancer cells [23]. During the last decade, CAR-T cell therapy has been recognized as a promising immunotherapy against hematological malignancies. Several CAR-T cell therapies, targeting molecules such as CD19, CD20, CD22, and BCMA (B-cell maturation antigen), have been either approved or have completed clinical validation trials and have proved to be a revolution in the treatment of hematological malignancies [24,25,26,27]. CD19-specific CAR-T cells with CD28/CD3ζ dual-signaling caused a significant improvement in acute lymphoblastic leukemia (ALL) and chronic lymphocytic leukemia (CLL) [28,29], signifying the promising antitumor efficacy of CAR-T therapies in human cancers. Presently, efforts are being made to further optimize CAR-T cell therapy, considering the CAR design, transduction and cell culture procedures [30,31]. However, CAR-T cell therapy has secured limited success in solid tumors, due to the lack of specific antigens, target diversity, tumor heterogeneity and complexity of the tumor microenvironment [32,33,34].

The immune checkpoint (IC) molecules are involved in the coinhibitory signaling pathways of immune cells to maintain tolerance, which is used by cancer cells to escape immunosurveillance [35,36]. Immune checkpoint inhibitors (ICIs) are promising immunotherapies that strengthen the antitumor immune responses. ICIs suppress the coinhibitory signaling pathways and enhance the immune elimination of tumor cells [37,38]. ICIs have shown promising results in cancer immunotherapies, due to broad bioactivity, stable responses and efficacy in metastatic and chemotherapy-resistant tumors [39]. High expression of ICs and their ligand interaction in the tumor microenvironment (TME) significantly abolish the induction of an effective anti-cancer response and alter T cell-mediated immune responses against tumor-associated antigens (TAAs) [40]. Cytotoxic T lymphocyte-associated protein-4 (CTLA-4), programmed cell death protein-1 (PD-1), and programmed death ligand-1 (PD-L1) have been widely implemented ICs. Therefore, inhibitors against these ICs are the most prominent ICIs deployed so far [41]. T cell activation is inhibited by its surface-expressed CTLA-4 in the anti-cancer immune response [42,43]. The blockade of CTLA-4 by a monoclonal antibody (ipilimumab) leads to a persistent response of the T cells, causing depletion of the regulatory T cells (Tregs) from the TME and improving immune-responsive effects for cancer therapy [44,45,46].

PD-1 is also expressed on activated T cells and causes the inhibition of the signaling for antigen recognition on the TCR [47]. PD-1 and its ligand PD-L1 have been recognized as ICs [48]. PD-L1 is expressed in several cell types, including tumor, immune, epithelial, and endothelial cells. PD-L1 is involved in immune tolerance, autoimmunity, and fighting against infections and tumors [49]. Interferon-γ (IFN-γ) exposure increases PD-1 expression, which results in tumor cells escaping from the immunosurveillance exerted by T cells [50,51]. The monoclonal antibodies against PD-1/PD-L1 (e.g., pembrolizumab, nivolumab, cemiplimab, atezolizumab, avelumab, and durvalumab) have been approved by the FDA for cancer immunotherapies [52]. However, tumor resistance to ICIs and ICI-mediated toxicities are major obstacles in their applications for cancer immunotherapies [53,54].

### 1.2. Tumor Immune Microenvironment

Genetic/epigenetic modifications and dynamic crosstalk of the components of the tumor microenvironment (TME) greatly influence tumor formation and progression [55]. Tumor cells induce alterations in the molecules, cells, and other physical conditions of the host tissues. The TME comprises an extracellular matrix (ECM) as well as cellular and non-cellular components. The cellular components include immune cells, fibroblasts, vascular endothelial cells, pericytes, stromal cells, and adipocytes, whereas non-cellular components include cytokines, chemokines, growth factors, enzymes, and proteoglycans embedded in the ECM [56]. During tumor advancement, cancer and stromal cell interactions may lead to an altered immunophenotype adoption. The TME enhances angiogenesis for the transport of nutrients/oxygen and the removal of metabolic waste, thereby avoiding hypoxic and acidic microenvironments. Different innate and adaptive immune cells, including both pro- and anti-tumor cells, infiltrate into the TME. Although TME varies among different cancer types, it is highly complex and dynamic. Various components of the TME interact according to signals generated by surrounding cells and extracellular receptors of target cells. Accurate communication, organization, and homeostasis are vital for the development of neoplastic tissue. In the early stages of tumor growth, cancer cells develop a mutual relationship with the TME. Cancer cells deploy a complex signaling cascade controlling the functions of TME components to promote tumorigenesis and cancer metastasis. This leads to improved tumor cell survival and proliferation, whereas a poor response to cancer therapy and multi-drug resistance. Therefore, understanding the functions and mechanisms of the intercellular interactions is crucial to hinder the pathways resulting in cancer development [56,57,58].

#### 1.2.1. Immune Cells

As an important part of the TME, innate and adaptive immune cells can either inhibit or promote tumor growth. Chronic infection resulting in persistent inflammation is a common phenomenon leading to tumor formation in several types of cancer, such as gastric cancer, cervical cancer, and hepatocellular carcinoma [59]. T and B cells influence tumorigenesis. T cells are involved in recognizing tumor antigens, killing tumor cells, and secreting IFN-γ to suppress angiogenesis and IL-2 to enhance anti-tumor immune responses [60]. B cells produce antibodies against tumors. Natural killer (NK) cells are involved in the direct killing of tumor cells or blocking metastasis. Neutrophils modulate tumorigenesis by releasing cytokines and reactive oxygen species to promote inflammation and tumor cell apoptosis in the TME. However, immune cells can also be pro-cancer, like regulatory T cells (Tregs), which are anti-inflammatory, inhibiting anti-cancer responses and promoting tumor progression. Similarly, regulatory B cells (Bregs) promote tumor aggression and immune suppression. Although Tregs and Bregs primarily perform immunosuppressive functions, they can also exert anti-tumor effects under certain circumstances and reduce inflammation-driven carcinogenesis. This may be attributed to the functional heterogeneity of Tregs and Bregs [61,62]. Some tumor types are heavily enriched with macrophages and can reach 50% of a tumor’s mass. These macrophages cause hypoxia and secretion of cytokines in the TME, which promote angiogenesis and cancer development. Neutrophils can promote tumor growth by modifying the ECM to enhance angiogenesis and tumor progression. Cancer cells engage the neighboring stromal cells, such as vascular endothelial cells, fibroblasts, adipocytes, and stellate cells, to promote angiogenesis and tumor proliferation, invasion, and metastasis [60,63,64,65].

#### 1.2.2. Hot and Cold Tumors

Immune cells infiltrate into the TME in varying strengths, resulting in different tumor immunophenotypes, namely hot, cold, or immune exclusion. High infiltration of immune cells and inflammatory signals are characteristic of hot tumors, whereas limited infiltration of immune cells and inflammatory signals are characteristic of cold tumors. Immune exclusion tumors are characterized by immune cells at their periphery or within stromal tissues. Hot tumors respond to anti-PD-1 or anti-PD-L1 immunotherapy due to a high infiltration of PD-1-positive T cells [66]. Immune cell exclusion in cancer is caused by a multitude of factors, including physical barriers (ECM), abnormal tumor blood vessels, and immunosuppressive cells and molecules. Transforming growth factor β (TGF-β) and vascular endothelial growth factor (VEGF) play crucial roles in ECM remodeling, tumor vascularization, immune cell recruitment, and induction of immune suppression in the TME. TGF-β and VEGF have been targeted to convert cold tumors into hot tumors and enhance anticancer efficacy [67,68,69].

#### 1.2.3. Extracellular Matrix (ECM)

ECM, composed of collagen, fibronectin, elastin, and laminin, is an important molecular component of the TME. In solid tumors, the ECM makes up to 60% of the tumor mass. The aforementioned non-cellular components of the ECM play several important roles, such as the dissemination of biomechanical signals for the modulation of tissue morphological and homeostatic differentiation and the maintenance of a healthy dynamic correlation among the cells and the microenvironment. Normal crosstalk is disrupted by massive, altered signals in the TME, leading to abnormal cell behavior during tumorigenesis [70,71,72]. ECM remodeling is a tightly regulated homeostatic process, whereas in tumorigenesis, it can act as a tumor promoter or inhibitor. Therefore, the complexity and heterogeneity of the ECM pose challenges to selective therapeutic approaches across tissue and tumor subtypes. In particular, the activation/upregulation of alternate ECM components/pathways, suppression of immune responses and off-targeting hinder ECM-targeted therapies [73].

#### 1.2.4. Hypoxia and Immune Escape

Sufficient blood, nutrient, and oxygen supply is crucial for maintaining normal and cancer cells alive within tissues. The usual oxygen pressure varies among different organs of the body [74,75]. However, in the condition of ischemia, the oxygen partial pressure decreases due to underdeveloped or blocked blood vessels, leading to hypoxia. Defective tumor angiogenesis causes insufficient blood supply and oxygen to the highly proliferating cancer cells, creating a condition of hypoxia [76].

Low oxygen concentration influences the proliferation and function of T cells in vitro [77]. Lymph nodes and spleen are major regions of hypoxia in the body [78]. Hypoxia blocks CD8+ T cell activation through the stabilization of hypoxia-inducible factor (HIF1-α) and the suppression of TCR-mediated Ca^2+^ signaling [79]. HIF1-α has been shown to regulate the balance between Treg and T helper type 17 (Th17) differentiation. It also promotes PD-L1 expression on tumor cells, which causes suppression of the effector T cells (Teff cells), and modulates the responses of NK cells that allow tumor cells to escape from NK-mediated killing [80,81,82]. Hypoxia causes the upregulation of Treg cells by several mechanisms and inhibits the responses of Teff cells. Hypoxia promotes angiogenesis, tumor tolerance, and skews the CD4+ cells towards a Th2 phenotype, resulting in IL-4-induced macrophage polarization [83,84]. Hypoxia causes the reduction in IFN-γ, IL-2 and T cells in vivo. Hypoxia generates reactive nitrogen species, which cause nitration of the TCR and CD8+ cells, thus reducing their capability to recognize the MHC-displayed antigen [85,86]. Hypoxia increases the infiltration of TAMs to reduce inflammation and promote tumor progression by angiogenesis and matrix remodeling [87,88]. During high tumor invasion and hypoxia, HIF1-α promotes neutrophil survival and stabilization, maintaining elevated levels of neutrophils with defective apoptotic capabilities [89]. In the hypoxia condition, HIF1-α regulates several features of DCs, including activation, maturation, antigen presentation, and expression of CD80 and CD86 co-stimulatory molecules [90].

#### 1.2.5. Acidosis and Immune Modulation

The high fermentative carbohydrate metabolism and poor perfusion cause the low pH of tumor acidosis found in most cancer types [91,92,93]. The increased glycolysis and high lactic acid level promote acidification of the TME, thus greatly influencing tumor immunity and overcoming immune surveillance [94]. Acidosis greatly influences carcinogenesis by somatic selection of cancer cells for prolonged cell survival, diversion from apoptosis, and drug resistance [95]. Acidosis also modifies the functions of immune cells such as T cells, neutrophils, macrophages, and DCs [96]. Cancer cells mostly depend on glycolysis due to alterations in energy demands for cell division, growth, and migration [97]. Thus, elevated glycolysis greatly depletes glucose availability, causing the death of activated T cells in the acidic environment [98]. However, acidic TME does not affect CD4+ Treg cells, due to their prime reliance on fatty acid oxidation [99]. In tumor-specific CD8+ T cells, acidosis decreases cytolytic activity and cytokine secretion [100]. Reversing the acidic pH can revert T cell anergy in vitro; however, prolonged acidic pH could permanently damage T cells or activate T cell apoptosis. Acidosis also stimulates the inflammatory response of endothelial cells, enhances leukocyte infiltration, and increases cancer cell metastasis [101,102,103,104,105,106,107]. Moreover, tumor acidosis increases the expression of PD-L1 in cancer cells as an immune escape mechanism [108,109]. Targeting tumor-associated acidity has been evaluated as a promising approach to augment the effects of immunotherapy in preclinical cancer models [110].

The complex TME is highly dynamic in different types of cancers and various progression stages of cancer, creating complications in anti-cancer therapies. The present discussion focuses on the immunotherapies for non-small cell lung cancer (NSCLC) and their associated complications of immune-related adverse events (irAEs).

## 2. Immunotherapy in Non-Small Cell Lung Cancer (NSCLC)

### 2.1. ICIs in NSCLC

Lung cancer is the major cause of cancer mortality in the world [111]. NSCLC is the most common type, accounting for over 80% of all lung cancers. Adenocarcinoma is the most common histologic subtype, accounting for about two-thirds of all NSCLC, and the other third has squamous cell histology. Adenocarcinoma is enriched with driver mutations in genes, the most common of which are EGFR (epidermal growth factor receptor), KRAS (Kirsten Rat Sarcoma), BRAF, and ALK (anaplastic lymphoma kinase). These gene mutations are less frequent in the squamous histology. These mutations could lead to an unconducive TME for immune activation, depletion of certain anti-tumor immunity, increased influx of Tregs and other immune-suppressive cells/molecules, and activation of immunotherapy resistance pathways [112]. Furthermore, the expression of the immune checkpoint protein PD-L1 varies across these tumor histological subtypes. PD-L1 expression is denoted by the tumor proportion score (TPS), which measures the percentage of viable tumor cells expressing PD-L1 in NSCLC. TPS < 1% denotes low expression and TPS > 50% denotes high expression. PD-L1 TPS is a predictive biomarker for response to immunotherapy with PD-1 inhibitors nivolumab, pembrolizumab, cemiplimab, and PD-L1 inhibitors atezolizumab and durvalumab [113,114]. However, studies have also shown therapeutic benefits with TPS < 1% expression. PD-L1 inhibitors are less effective in patients with specific oncogenic driver mutations, such as EGFR exon 21 L858R mutations, EGFR exon 19 deletions, and ALK rearrangements. In patients with these mutations, first-line chemoimmunotherapy reported inferior outcomes [115,116].

The clinical efficacy of cancer treatments is assessed with a computed tomography (CT) or positron emission tomography (PET) scan every 4–6 cycles of therapy to assess and measure tumor response according to the standard set by the Response Evaluation Criteria In Solid Tumors (RECIST) [117,118]. However, patients receiving immunotherapy may develop atypical patterns of immunotherapy response termed pseudo-progression. In pseudo-progression, the collective longest diameter of the target lesion is at least a 30% increase from the time of disease progression determination, but not from the baseline per RECIST criteria. The usual reason for the pseudo-progression is the infiltration of immune cells into the tumor within the first three months of immunotherapy. Pseudo-progression has been reported in about 3 to 5% of patients [119]. Therefore, careful considerations are needed for the assessment of tumor response to immunotherapy alongside not abandoning treatment prematurely.

Treatment of NSCLC with immunotherapy has significantly improved patient survival and changed the treatment paradigm for NSCLC over the past decade [120,121]. Immunotherapy is approved for metastatic cancer as a first-line single agent or in combination with chemotherapy, in the adjuvant setting following surgery, chemotherapy and radiation, and now in the neoadjuvant setting for early-stage resectable tumors.

### 2.2. Early-Stage Resected Tumor Adjuvant Chemoimmunotherapy

For early stage-I NSCLC, the therapy of choice is a lobectomy, segmentectomy, or wedge resection of the tumor. Alternatively, if patients are not surgical candidates, they should be treated with stereotactic body radiation therapy (SBRT) to the lesion. Immunotherapy has now played a vital role in the perioperative period with neoadjuvant therapy and adjuvant therapy for those stage II-IIIA resected NSCLC.

The first trial (IMpower010) for adjuvant chemotherapy in patients with resected stage II-IIIA NSCLC that had non-squamous and squamous histology and PD-L1 > 1% reported a disease-free survival benefit with atezolizumab (1200 mg every 21 days; for 16 cycles or 1 year) in comparison to best supportive care (observation; regular scans for disease recurrence) [122].

The Keynote 091 trial showed improved disease-free survival (DFS, 53.6 months vs. 42 months, *p* = 0.0014) in patients with resected stage IB (tumors ≥ 4 cm in diameter), II, or IIIA NSCLC, regardless of PD-L1 status, receiving pembrolizumab adjuvant immunotherapy for 12 months [123].

### 2.3. Neoadjuvant/Perioperative Immunotherapy

Benefits of neoadjuvant therapy include increasing operability, early eradication of micrometastasis, and assessment of response to treatment with a major/complete pathologic response associated with improved 5-year overall survival (OS) and DFS rates [124]. The use of neoadjuvant immunotherapy is proposed to be more beneficial than adjuvant therapy due to the larger antigen presented by the primary tumor, which can then lead to increased antigen presentation and T cell priming with the use of immunotherapy. This has been evaluated in multiple trials.

Nivolumab was the first immunotherapy approved for neoadjuvant treatment in resectable stage IB (≥4 cm)-IIIA NSCLC, based on the benefits seen in the CheckMate 816 study. Results from the study showed significantly longer EFS and higher rates of complete pathological responses with chemotherapy plus nivolumab vs. chemotherapy alone [125]. The addition of nivolumab to neoadjuvant chemotherapy did not increase the incidence rate of adverse events or reduce the feasibility of surgery [125].

CheckMate 77T showed event-free survival benefit of perioperative neoadjuvant nivolumab plus chemotherapy in resectable IIA-IIIB NSCLC. The percentage of patients with 18-month event-free survival was 70.2% in the nivolumab plus chemotherapy group and 50.0% in the chemotherapy group [126].

The open-label phase II NADIM II trial showed higher rates of pathological complete responses, progression-free survival (PFS) and OS in resectable stage IIIA and IIIB with perioperative platinum doublet with nivolumab vs. chemo alone [127].

The NEOSTAR trial evaluated the use of ipilimumab together with nivolumab combination (dual CTLA-4 and PD-1 antagonists), which showed an increased pathological response in comparison to single-agent nivolumab [128].

The Keynote 671 trial evaluated the benefit of perioperative pembrolizumab plus platinum doublets vs. chemotherapy alone in stage II, IIIA, IIIB resectable NSCLC, and reported significant improvement in event-free survival, major and complete pathological response and overall survival [129].

The Aegean study evaluated perioperative durvalumab with platinum doublet in resectable NSCLC Stage II to Stage IIIB N2 disease with increased 12-month event-free survival (EFS) and increased pathological complete response (CR) [130].

The Neotorch trial evaluated the perioperative toripalimab plus chemotherapy vs. placebo plus chemotherapy in patients with stage II or III resectable NSCLC and showed a significant improvement in event-free survival (HR 0.40; *p* < 0.001) in the toripalimab + chemotherapy arm compared to chemotherapy alone [131].

It is now standard to consider patients with resectable stage IB to IIIA for perioperative chemo-immune therapy for fit patients who have resectable NSCLC, regardless of PD-L1 status and histology subtype except the caveat that patients with EGFR mutations or ALK fusions are generally excluded from or derive less benefit from perioperative immunotherapy [132].

### 2.4. Locally Advanced or Metastatic Setting

For patients with good performance status and curable, locally advanced/unresectable stage III NSCLCs, standard therapy is chemotherapy platinum doublet combined with concurrent radiation therapy followed by maintenance with durvalumab per the PACIFIC trial [132,133]. The stage III unresectable NSCLC patients received definitive concurrent radiation therapy and platinum doublet but did not progress and were randomized to consolidation therapy with durvalumab. The addition of durvalumab for 12 months vs. placebo resulted in significantly improved PFS (16.9 vs. 5.6 months) and median OS (47.5 vs. 29.1 months) [132].

First-line therapy for metastatic disease is guided by the driver mutational profile and the PD-L1 TPS expression. Patients with driver mutations, such as the EGFR and ALK mutations, respond with improved survival to a targeted agent rather than traditional chemotherapy and radiation. However, in the majority of NSCLC without driver mutations, the best choice is chemotherapy in combination with immunotherapy, whereas patients with PD-L1 TPS > 50% may be eligible for single-agent immunotherapy.

Single-agent immunotherapy first-line vs. chemotherapy was evaluated in multiple trials. Keynote 024 studied pembrolizumab vs. platinum doublet in patients with TPS ≥ 50% and the median overall survival (OS) was improved from 13.4 months in the chemotherapy group vs. 26.3 months in the pembrolizumab group [134,135]. IMpower 110 studies also showed improved median OS (20.2 vs. 13.1 months) with atezolizumab vs. chemotherapy in patients with PD-L1 TC > 50% [136]. EMPOWER-Lung-1 treatment of unresected IIIB, IIIC and Stage IV NSCLC with PD-L1 TPS 50% or more, with cemiplimab, showed an improved median overall survival of 12.8 months in comparison to chemotherapy (26.1 vs. 13.3 months) [137].

Keynote 189 showed that treatment of metastatic non-squamous NSCLC patients without EGFR and ALK mutations with a combination of platinum chemotherapy plus pemetrexed with pembrolizumab resulted in increased response rates and OS across all subgroups of PD-L1 expression. The 5-year OS rates were 19.4% versus 11.3% between the pembrolizumab plus platinum-pemetrexed cohort and the placebo plus platinum-pemetrexed cohort [138].

Keynote 407 reported that squamous type NSCLC treatment with carboplatin doublet with paclitaxel with/without pembrolizumab for four cycles followed by maintenance pembrolizumab resulted in improved PFS of 6.4 months vs. 4.8 months and median OS of 15.9 vs. 11.3 months with all subgroups of PD-L1 TPS expression [139].

The IMpower 130 trial evaluated atezolizumab plus chemotherapy (carboplatin and nab-paclitaxel) and chemotherapy alone in stage IV non-squamous NSCLC patients without EGFR and ALK mutations. Compared to chemotherapy alone, the atezolizumab plus chemotherapy group showed an increase in the PFS (7.0 vs. 5.5 months) and median OS (18.6 vs. 13.9 months) [140].

IMpower 150 showed that the carboplatin doublet with atezolizumab and bevacizumab (VEGF inhibitor) in the metastatic NSCLC (no EGFR or ALK alterations) in comparison to doublet with bevacizumab resulted in improved median OS (19.5 vs. 14.7 months) [141].

Checkmate 227 resulted in the approval of a dual immune checkpoint with ipilimumab and nivolumab due to the improved median OS of 17.1 months vs. 13.9 months compared to chemotherapy in patients with advanced NSCLC in all patients (combined PD-L1 ≥ 1% and <1%) [142].

Checkmate 9LA found that chemotherapy with ipilimumab and nivolumab vs. chemotherapy alone resulted in improved median OS to 15.6 months vs. 10.9 months in patients with stage IV or recurrent NSCLC [143].

The Phase III POSEIDON trial showed significantly improved PFS (6.2 vs. 4.8 months) and OS (14.0 vs. 11.7 months) with durvalumab plus tremilimumab plus chemo vs. chemo alone and without any new significant tolerability issues [144]. POSEIDON and checkmate 9LA revealed benefit in tumors harboring mutations like STK11 and KEAP1, which are associated with poor outcomes [145]. Exploratory subgroup analyses from these trials have suggested potential benefit in STK11/KEAP1-mutated tumors, but these are hypothesis-generating subset analyses, not definitive evidence. There is ongoing prospective randomized trial analysis from TRITON study to test this hypothesis [146].

On the other hand, the MYSTIC trial reported that the dual ICI durvalumab and tremelimumab for blocking PD-L1 and CTLA-4 resulted in increased toxicity and did not improve outcomes vs. chemo alone [147].

In addition to PD-1/PD-L1 and CTLA-4 inhibitors, therapeutics have been developed to target other immune checkpoints such as LAG-3 and TIGIT. The RELATIVITY-104 randomized phase II trial demonstrated that relatlimab, a LAG-3 blocking antibody, in combination with nivolumab and chemotherapy improved PFS and OS in metastatic non-squamous NSCLC patients with PD-L1 ≥ 1% tumor subgroups [148]. Based on these results, RELATIVITY-1093, a phase III study, is currently underway to compare OS of relatlimab plus nivolumab and platinum-doublet chemotherapy (PDCT) to pembrolizumab plus PDCT in patients with metastatic non-squamous NSCLC and tumor PD-L1 expression ≥ 1% [149]. Tiragolumab is a monoclonal antibody targeting TIGIT. The phase III randomized SKYSCRAPER-06 trial showed that tiragolumab plus atezolizumab and chemotherapy did not improve PFS or OS, compared with pembrolizumab plus chemotherapy, in patients with advanced non-squamous NSCLC [150].

### 2.5. Radiation Therapy and ICI

Radiation therapy (RT) has been proven to be a suitable cure for non-surgical patients with early-stage NSCLC. RT combined with chemotherapy showed promising results in unresectable stage-III patients as well as symptomatic local control of metastasis. RT is beneficial in combination with immunotherapy, with an improvement in overcoming resistance to radiation and immunotherapy. RT can modify the TME by increasing IFN release, which stimulates the complement system for the induction of immunogenic cancer cell death [151,152]. RT improves the priming of CD8+ T cells and upregulates PD-L1 expression on the surface of tumor cells [152]. RT combined with systemic ICI can lead to systemic immune tumor control known as the abscopal effect [153]. However, there exists a high risk of increased toxicity, and increased irAE cases are reported with concurrent use, particularly pneumonitis [154].

RT with fewer than 5 fractions and smaller fields is superior to larger fields with greater than 15 fractions in the ability to unleash the abscopal effect. A pooled clinic trial (phase-II) using a combination of pembrolizumab and radiation in metastatic NSCLC showed an improved overall response rate, particularly in the low PD-L1 TPS group [155,156]. Thereby, the data support the notion that RT can promote immunogenic activation, inflammation, and improved response to ICI [157].

### 2.6. CNS Disease and ICI

Among central nervous system (CNS) diseases, brain metastasis is one of the direst consequences of metastatic NSCLC, complicated by cerebral edema, motor sensory deficits, seizure disorders, and loss of vision and the ability to speak. Reports showed that up to 45% of patients with NSCLC will develop brain metastasis through the course of their disease [158].

EGFR and ALK mutant NSCLC are known to have excellent therapy with blood–brain barrier (BBB)-penetrating drugs such as osimertinib and alectinib. However, a large portion of patients lack these driver mutations. Evidence for systemic therapy is limited, as most pivotal trials excluded patients with untreated brain metastasis. Analysis of Keynote 001, 010, 024 and 042 trials showed that pembrolizumab systemic therapy enhanced OS in patients with PD-L1 expression > 50% [134,135,159,160]. Keynote 021, 189 and 407 trial analyses showed that pembrolizumab combined with chemotherapy has improved outcomes in patients with stable/treated brain metastases in all PD-L1 expression subsets [138,161,162]. Among the treated brain metastases group of the Checkmate 9LA trial, dual ipilimumab and nivolumab and chemotherapy improved OS of patients [163,164]. The same was observed in the Checkmate 227 trial among the subgroup analysis of treated brain metastases patients [142]. In the phase II trial of brain metastasis patients, the Atezo-Brain trial evaluated 40 advanced non-squamous NSCLC patients with untreated brain metastases. The intracranial response rate was 42.7% and the systemic response rate was 45%, with a median OS of 11.8 months, when treated with atezolizumab plus carboplatin and pemetrexed [165].

Historical analysis recommends upfront systemic therapy in patients with small asymptomatic intracranial disease. However, for patients with symptomatic disease, cranial irradiation and cautious use of anti-PD-L1 therapy concurrently are recommended. This is due to the reported increased risk of radiation necrosis, and concern of reduced effectiveness of immune checkpoint blockade due to the use of systemic corticosteroids (yet to be evaluated prospectively in clinical trials). Additionally, studies have shown a correlation between NSCLC brain metastasis and alterations in the CDK4/CCND1, CDKN2A/2B, and PI3K signaling pathways as potential therapeutic targets [166].

In summary, major clinical trials of immunotherapy in different settings of NSCLC treatment are outlined in Table 1.

## 3. Immune-Related Adverse Events in Cancer Immunotherapy

**Figure 2:**
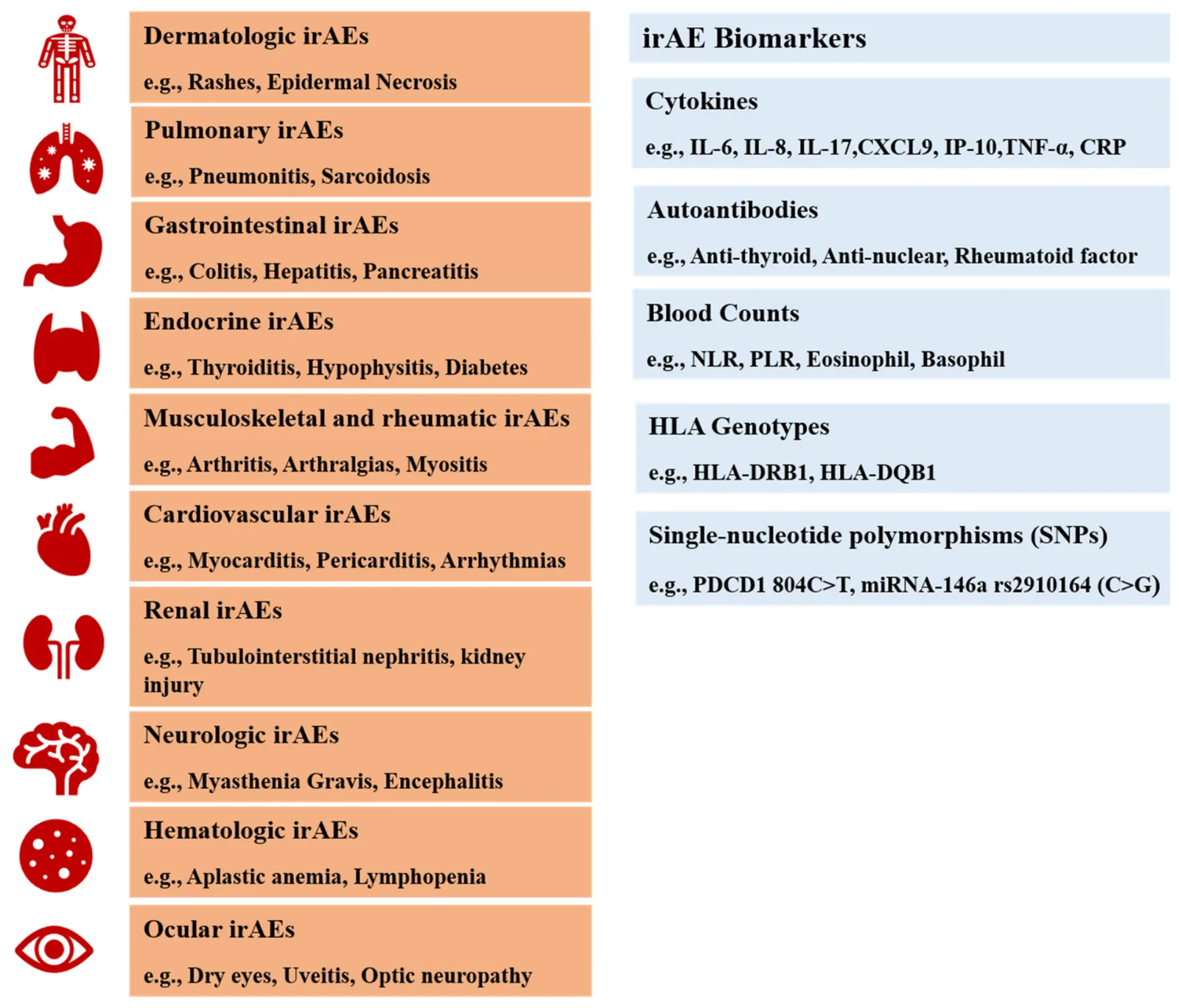
Immune-related adverse events (irAEs) and biomarkers.

Immune-related adverse events (irAEs) are inflammatory and autoimmune reactions that occur secondary to ICI therapy, with severity from mild to life-threatening, and can occur in almost any organ system (Figure 2) [167]. ICIs act to thwart tumor immune evasion via the inhibition of systems of self-tolerance. As a result, ICI irAEs often present similarly to de novo autoimmune disease [5,167]. irAE development may indicate treatment efficacy and some studies correlate the development of irAEs with improved progression-free survival (PFS) and overall survival (OS), but the association of irAEs with improved efficacy remains controversial as there are variable outcomes [168,169,170]. Despite the risk of developing irAEs, ICI monotherapy has demonstrated reduced toxicity profiles when compared to other standard modes of treatment including chemotherapy [171,172].

### 3.1. irAE Grading

irAEs are graded by severity by the Common Terminology Criteria for Adverse Events (CTCAE), a standardized scale developed by the National Cancer Institute (NCI). The CTCAE is a reporting system for all cancer research trials performed under the guidance of the NCI and allows for the comparison of irAEs from multiple systems. The CTCAE is regularly revised from time to time with improved and added guidelines based on clinical and patient outcomes (CTCAE Version-2, 1999, Version-3, 2006, Version-4, 2010, and Version-5, 2017). The CTCAE incorporated terminology from the Medical Dictionary for Medical Affairs (MedDRA), which had been another widely used reporting standard required by other research regulatory groups [173]. The CTCAE provides a numerical score according to grade severity and ranges from 1 (asymptomatic or mild presentation with symptoms that do not require medical intervention), 2 (moderate, with minimal, local, or non-invasive intervention indicated), 3 (severe or medically significant but not immediately life-threatening; hospitalization or prolongation of existing hospitalization indicated), 4 (life-threatening; urgent intervention indicated to prevent death), to 5 (death secondary to irAE development).

The presentation and development of irAEs differ depending on ICI agent class as well as monotherapy or combination therapy with other ICI classes [5]. The development of irAEs is correlated with improved treatment outcomes; however, the relationship with OS, PFS, or ORR based on the grade of irAE alone is more nuanced, noted to be better in low-grade and poorer in higher-grade irAEs [168,169]. The relationship between irAEs and clinical outcomes is not simple but very complex and involves several factors. Low-grade irAEs indicate persistent systemic immune activation, which in turn manifests in the form of robust anti-tumor activity. Severe irAEs, on the other hand, may lead to long-term interruptions or permanent discontinuations of treatments. Also, the use of high-dose corticosteroids and other immunosuppressants for irAEs may counter the effects of ICIs and lead to deleterious outcomes [174]. Management decisions are made based on several factors, including the grading of irAEs, status of disease, availability of alternative treatment options, and organs that are affected.

Risk factors associated with irAE development include specific HLA genotypes, increased BMI, elevated pre-treatment levels of pro-inflammatory cytokines, thymic tumors, and tumor mutation burden [175,176]. While irAE development can occur early during ICI treatment, a delayed immune response after ICI therapy has been discontinued is also possible and is likely secondary to prolonged receptor site occupancy [177].

### 3.2. Common Types of irAEs

Organ systems that most commonly develop irAEs during ICI therapy are dermatologic, gastrointestinal (GI), pulmonary, and other organ systems prone to idiopathic autoimmune disease development, such as the thyroid, adrenal, and pituitary glands [5,170,178]. While irAEs that result in death are rare, still 0.36–1.23% irAE incidence proves lethal. The most common irAEs leading to mortality during ICI treatment of NSCLC are pneumonitis, hepatitis and colitis [5,171,179].

#### 3.2.1. Dermatologic irAEs

Dermal toxicities are present in ICI treatment, with the average onset between 3 and 4 weeks post the initiation of treatment [175,177]. Although dermatological toxicity rarely results in grade 3 or greater irAEs, severe manifestations are significant and potentially life-threatening, including bullous rashes, toxic epidermal necrosis, and Stevens–Johnson Syndrome [175].

#### 3.2.2. Pulmonary irAEs

Pulmonary irAEs include pneumonitis and sarcoidosis. Pneumonitis is a broad term that can be used to describe any ICI-induced inflammatory lung disease and is associated with high morbidity and mortality [5,180]. Factors associated with pneumonitis development include tumor types, previous radiotherapy, pulmonary co-morbidities, and anti-PD-1 therapy [176,179]. The incidence of pneumonitis varies widely, with the highest reported incidence of 19% and among them, 12% showed a grade 3 or higher score [175,179,181]. Pneumonitis can be difficult to recognize, and its symptoms can mimic treatment sequelae and progression of the disease [171]. The most common symptoms of pneumonitis include dry cough and dyspnea, although other nonspecific symptoms, including chest pain and fever, are also common [171,176]. Definitive diagnosis of pneumonitis can be challenging due to nonspecific and varying features on X-ray and CT scans that range from consolidation to nodules indicating a sarcoid-like reaction [176]. Patients with lung cancer often have comorbid lung disease, which can further confound image results and delay diagnosis. Sarcoidosis is usually asymptomatic and rare, making its diagnosis relatively difficult. While it can occur in numerous locations throughout the body, studies indicate that it occurs in the lungs 60% of the time and is often mistaken for disease progression. As a result, sarcoidosis should remain in the differential diagnosis whenever nodular lesions are identified and biopsy is recommended [175].

A systematic review shows that the incidence of tuberculosis (TB) is increased in cancer patients treated with PD-1/PD-L1-based immunotherapy when compared to the general population [182]. Studies also demonstrate that the use of ICIs is associated with TB reactivation, latent TB infection, and increased TB risk in cancer patients [183,184]. Screening and monitoring for TB reactivation and latent TB infection may be beneficial for cancer immunotherapy patients with prior history or from TB-endemic areas.

#### 3.2.3. Gastrointestinal (GI) irAEs

Gastrointestinal irAEs, apart from colitis, tend to be mild and include pancreatitis, hepatitis, cholecystitis, and cholangitis [175]. Among GI tract toxicity, the most common one is diarrhea in almost 50% of reported patients, and patients receiving CTLA-4 inhibitors reported higher rates of GI irAEs compared with other ICI therapies [5,177]. Diarrhea is the most common symptom of colitis, but other more severe symptoms include bowel perforation, bleeding, ischemia, necrosis, and toxic megacolon [175].

Hepatitis can present asymptomatically and should be suspected with elevations in serum AST and ALT. Hepatitis irAEs are more often associated with CTLA-4 inhibitor therapy and on average occur 15 weeks after initiation of therapy but can be delayed months to years after therapy discontinuation. Cholangitis and cholecystitis are extremely rare, associated with anti-PD-L1 therapy, and tend to be refractory to steroid treatment [175].

#### 3.2.4. Endocrine irAEs

Endocrine irAEs are significant due to the likelihood of permanent damage that often necessitates lifelong treatment, including hypophysitis, adrenal insufficiency, diabetes, and thyroid disorders [170,175,178,185].

Thyroid dysfunction is particularly associated with PD-1 inhibitors and the CTLA-4/PD-1 inhibitor combination therapy. The presentation of clinical symptoms varies from asymptomatic thyroiditis to thyrotoxicosis. It can be difficult to differentiate destructive thyroiditis from Graves’ disease and many patients who experience anti-PD-1 ICI-induced Graves’ disease are negative for anti-TSH antibodies [170]. The classic presentation of hypothyroidism includes thinning of hair and nails, cold intolerance, weight gain, lethargy, dry skin, and depression [186]. In severe cases, it can also present with psychosis, hypothermia, and stupor [186]. Hyperthyroidism, on the other hand, presents with anxiety, restlessness, heat intolerance, thinning of hair and nails, frequent bowel movements, hyperreflexia, and can even lead to angina and atrial fibrillation [187,188]. While studies agree that the incidence of all types of irAEs correlates with improved OS and PFS, some studies further suggest that, specifically in the treatment of NSCLC, endocrine toxicities such as thyroiditis indicate improved ICI efficacy and increased OS [169,170,189]. This trend in outcomes is particularly true of overt thyroiditis resulting in hypothyroidism, rather than subclinical thyroiditis [170,189]. While a consensus has yet to be reached on the significance of this data, the study results are promising and warrant further evaluation.

ICI-induced diabetes mellitus (DM) occurs in less than 1% of patients and is most often associated with anti-PD-L1 therapy. These patients present with type-1 diabetes mellitus (T1DM) and diabetic ketoacidosis but can also be asymptomatic [175].

ICI-induced hypophysitis is usually associated with anti-PD-L1 and PD-1 (in 1% patients) and anti-CTLA-4 therapy (in 4% patients) and is a result of immune cell infiltration into the pituitary [190]. ICI-induced hypophysitis is primarily associated with fatigue, headaches, nausea, vomiting, visual disturbances, pituitary enlargement, and gonadotrophic deficiency, such as erectile dysfunction and decreased libido [175,190].

ICI-induced adrenal insufficiency is most often secondary adrenal insufficiency and a result of ICI-induced hypophysitis. Even in patients without a history of hypophysitis, ICI therapy can damage the adrenal glands, resulting in primary adrenal insufficiency [170]. This condition is often difficult to diagnose as the symptoms are general and nonspecific, such as malaise, nausea, and hypotension [175]. However, it is important to have a low threshold for clinical suspicion due to the risk of developing vasodilatory shock [170,175].

#### 3.2.5. Musculoskeletal and Rheumatic irAEs

Musculoskeletal irAEs include arthralgias, arthritis, myositis, and polymyalgia rheumatica. Arthralgia and myalgia are commonly associated with cancer therapies such as radiation and chemotherapy and are therefore often misattributed. However, recognizing these irAEs is important as they are often symptoms of inflammatory arthritis and inflammatory myositis and can result in permanent joint damage. Polymyalgia rheumatica often presents with joint pain, stiffness, and an increased risk of developing giant cell arteritis [175].

#### 3.2.6. Cardiovascular irAEs

There are numerous cardiovascular irAEs, which include myocarditis, pericarditis, venous thromboembolism, and arrhythmias. Reported cases of myocarditis irAEs are steadily increasing in prevalence, likely due to increased ICI therapy utilization and improvement in irAE identification. The typical symptoms of ICI cardiotoxicity are chest pain and dyspnea [5,175].

#### 3.2.7. Renal irAEs

Renal system irAEs are confounded by acute kidney injury caused by the nephrotoxic medications and dehydration that may be encountered during treatment. Tubulointerstitial nephritis is the most reported renal irAE. Patients often present with elevated serum creatinine, proteinuria, and sterile pyuria [5].

#### 3.2.8. Neurologic irAEs

Neurotoxicities are numerous and include myasthenia gravis, Guillan–Barré, encephalitis, aseptic meningitis, and peripheral neuropathy [5,175]. Myasthenia gravis has the highest rate of mortality with a worse prognosis for patients with comorbid myositis or myocarditis [5]. Encephalitis often presents with confusion, nausea, fevers, agitation, and changes in speech. Most ICI-induced encephalitis is not associated with autoimmune antibody production, and its etiology is still uncertain [175,191].

Three rare but significant irAEs warranting particular attention and distinction are myositis, myocarditis, and myasthenia gravis [5,175]. These irAEs are concurrent or occur at different times during ICI treatment and often have similar signs and symptoms, including muscle weakness, diaphragm weakness, and restricted/weak ocular movement [175]. While these irAEs are rare, they present an elevated risk for mortality and warrant a low threshold for clinical suspicion.

#### 3.2.9. Hematologic irAEs

Hematologic irAEs are rare but potentially serious and include aplastic anemia, lymphopenia, neutropenia, acquired thrombotic thrombocytopenia purpura, with the most common being thrombocytopenia and autoimmune hemolytic anemia (AIHA) [175,190,192,193]. AIHA is mostly associated with anti-PD-1 agents and is usually responsive to steroid therapy, generally resolving within 2 weeks of treatment [175].

#### 3.2.10. Ocular irAEs

Dry eyes and uveitis are the most common ocular irAEs reported [175]. Other ocular irAEs are frequent sequelae from neurological or musculoskeletal irAEs, such as optic neuropathy, ocular muscle myopathy, ulcerative keratitis, and Vogt–Koyanagi–Harada (VKH) syndrome. Symptoms vary depending on the etiology and include conjunctival injection and inflammation of orbital structures and can lead to vision loss if not treated promptly [5,175].

### 3.3. Best Practice Guidelines for irAE Treatment

ICI-induced toxicities can occur in nearly every system of the body and thus require careful surveillance and prompt treatment. There is currently no prospective study data that describes the ideal treatment of irAEs [190]. Instead, best practice guidelines have been produced by several groups, including the American Society for Clinical Oncology (ASCO), the Society for Immunotherapy of Cancer (SITC), and the European Society for Medical Oncology (ESMO) [175,190].

Before the initiation of ICI therapy, patients should be educated on irAEs. Pretreatment testing should include obtaining baseline labs and EKG and troponin should be drawn on patients with a history of cardiac disease, diabetes, or whose treatment plan includes a combination of CTLA-4/PD-L1 inhibitors as these patients are at greater risk of myocarditis [175].

Treatment of specific irAEs depends on the grade and organ system affected. Although low-grade irAEs may serve as a reference for ICI efficacy monitoring, they should be managed according to established guidelines, as unaddressed irAEs may cause serious consequences. Generally, with all irAEs of grade 2 or higher, ICI treatment is held, and glucocorticoid therapy should be initiated except in cases of endocrine irAEs where hormone replacement is the management. An initial dose of 1–2 mg/kg/day of prednisone or methylprednisolone may be used for the management of grade 3 or higher irAEs [194]. In cases where irAEs are refractory to glucocorticoid therapy, immunosuppressive adjuncts such as methotrexate, mycophenolate mofetil, IL6 receptor inhibitors (e.g., tocilizumab), and TNF-α inhibitors (e.g., infliximab) may be used [175,177,190,195]. The anti-IL-6 receptor inhibitor tocilizumab has been noted to provide an antitumor effect after ICI therapy has been discontinued. Specialists should always be consulted for irAEs of grade 3 or greater, irAEs refractory to treatment, if hospitalization is required, or if the irAE is neurological or rheumatological in nature. If ICI therapy is discontinued due to the development of grade 2–3 irAEs, rechallenge may be considered after thorough risk–benefit analysis as a measure of shared decision-making between the patient and physician [175].

### 3.4. Consideration for Patients with Autoimmune Disease

While pre-existing autoimmune disease (AID) is not an absolute contraindication for ICI therapy, consideration should be given to the benefits and risks associated with ICI treatment and AID flares [175,196,197]. The past trials did not include patients with pre-existing AID; however, recent studies showed promising outcomes for the safe use of ICI treatment in patients with AID and demonstrated the development of predominantly low-grade irAEs [197,198,199]. More prospective studies are warranted to better understand the risks and best practices of treating AID patients with ICI therapy.

### 3.5. ICI Rechallenge Considerations

Patients who complete therapy with treatment-resistant tumors or who need to discontinue ICI therapy after experiencing a grade 2 or greater irAE are common candidates for ICI therapy rechallenge. Presently, no standardized rechallenge guidelines are available, and the existing data is limited (small sample size). It has been shown that patients with a lower Eastern Cooperative Oncology Group (ECOG) score and longer length of initial treatment are more likely to tolerate ICI rechallenge, possibly due to the patient’s overall health and ICI treatment tolerance [200]. CTLA-4 inhibitors carry a higher risk of irAE recurrence compared to PD-1/PD-L1 inhibitors [201]. Studies demonstrated a 28.8% irAE recurrence rate in patients initiating rechallenge, with colitis, hepatitis, and pneumonitis being more likely than other irAEs to recur. However, these recurrences tend to be less severe than the initial irAE [167,200,202]. Some studies have attempted to mitigate irAE recurrence and increase ICI therapy tolerance by utilizing a different ICI therapy class than the initial treatment or only utilizing a single treatment class. However, results of these trials have been inconsistent and inconclusive [200]. A systematic review by Zhao et al. found that ICI rechallenge had a higher risk and overall equivalent efficacy to the initial treatment [203].

### 3.6. Proposed Mechanisms of irAEs

Currently, a definitive mechanism of irAE development remains unclear. Some researchers propose that irAEs may possibly develop due to the pre-existing, yet undetected, subclinical inflammation that could flare by the inhibited T-cell self-tolerance mechanisms [169]. Other mechanisms include uninhibited macrophages causing cytotoxic damage and the activation of B cells that then produce autoantibodies [202]. PD-L1 expression is relatively higher in the thyroid than other tissues and PD-1 inhibitors have been linked to destructive thyroiditis. ICI-induced thyroiditis is likely caused by activation of T-lymphocytes that initiate the destruction of otherwise healthy thyroid tissue [170,204]. Shared antigens between target tumor cells and tissues have also been implicated in irAE development, and in one case study, patients experiencing myocarditis irAEs were found to have shared antigens in skeletal, cardiac, and tumor tissue [5]. Molecular pathways involved in cytokine signaling, such as TNF-α, IL-6, IL-1β, IL-17, and IFN-γ, have been shown to play important roles in irAEs. Interventions targeting the cytokine pathways, such as infliximab and tocilizumab, are used to mitigate complications associated with irAEs [205].

### 3.7. Microbial and Viral Influences

The gut microbiome and the bacterial taxa are closely associated with ICI effectiveness and irAEs, where some bacterial phyla showed improved outcomes, while others have contributed to poorer outcomes [206]. A microbiome profiling study using 16S ribosomal RNA gene sequencing and metagenomic analyses revealed correlations between the ICI responsiveness and bacterial taxa including species of the *Bacillota* (*Firmicutes*) phylum, *Bacteroidota* phylum, *Actinomycetota* phylum, *Collinsella aerofaciens* and *Verrucomicrobiota* phylum [207].

In melanoma patients treated with anti-PD-1 immunotherapy, varied gut microbiome compositions were observed among the responding and non-responding patients. A group of 26 metastatic melanoma patients treated with Ipilimumab, where the gut microbiota enriched with *Faecalibacterium* and other members of the *Firmicutes* phylum, showed improved PFS and OS. Additionally, these patients experienced higher rates of ICI-induced colitis compared to patients who were not enriched with *Faecalibacterium* [169]. Another patient group enriched with *Bifidobacterium* species showed increased ICI response in renal cell carcinoma [207].

Patients enriched with taxa, such as *Bacillota*, *Bacteroidaceae*, and *Pseudomonadota*, showed shorter PSF or non-responsiveness to ICI immunotherapy. These predictor microbiomes in the gut promote a pro-inflammatory state and reduce the gut’s protective capabilities. *Pseudomonadota* causes the predisposition to ICI-induced colitis and tissue damage upon T-cell activation by inflammatory cytokines [207].

Viruses have also been implicated in irAE development with ICI treatment. While the overall significance has not been robustly examined, some case studies have identified a link between memory T-cell activation against dormant EBV antigens and ICI-induced encephalitis. In one study, non-acute encephalitis developed because of memory T-cell activation against EBV antigens present in both neural tissues and metastasized tumor tissues in the brain [191].

### 3.8. irAE Biomarkers

The predictive biomarkers of irAEs are crucial to identifying risk and reducing morbidity and mortality. This has proven difficult, however, as many of the mechanisms of irAEs are not fully understood and the use of ICI therapy as a clinical tool for disease treatment is a relatively new development.

Definitive tests for the prediction of specific irAEs are not available, but studies have defined trends that help predict irAE risk. Blood counts, autoantibodies, cytokines, HLA genotypes, and gene expression have served as predictive tools for irAEs [202].

#### 3.8.1. Serum Cytokines and Autoantibodies

A significant rise from baseline of cytokines such as IL-6, IL-8, CXCL9, and IP-10 has been associated with increased risk for irAE development [178,180,202,208,209,210]. In anti-CTLA-4 therapy, elevated IL-17 showed a correlation with the development of diarrhea and colitis, and down-trending IL-17 is an indication of resolution of the irAE. However, this trend was not noted in anti-PD-L1 therapies [208]. Studies also showed that elevated CRP levels were commonly associated with irAEs [180,209,211].

Anti-thyroid, antinuclear antibodies (ANAs) and rheumatoid factor (RF) have been correlated with an increased risk of irAE development [202]. Patients with autoantibodies at baseline are more likely to develop AID after initiation of ICI therapy [208].

#### 3.8.2. Blood Counts

Trends have been identified indicating that lower neutrophil to lymphocyte ratio (NLR) and platelet to lymphocyte ratio (PLR) correlate to increased risk of irAE development, whereas increased eosinophil and basophil counts are correlated to risk of developing dermal and endocrine irAEs [202]. High NLR has been correlated with decreased OS and may be an indication of poor response to ICI therapy [208].

#### 3.8.3. HLA Genotypes

While no single specific HLA is associated with all types of irAE development, studies linked specific HLAs with specific irAEs, such as HLA-DRB1 and HLA-DQB1, have been correlated with the development of dermatologic pruritus and colitis, respectively [212].

#### 3.8.4. Single-Nucleotide Polymorphisms (SNPs)

Studies have identified SNPs that modulate (increase or reduce) the risk of irAE development. Many of these genes are known to be linked to AID development and immune system modulation [202]. Some SNPs, such as the PDCD1 804C>T SNP (rs2227981), are linked to fewer irAEs [213], while the rs2910164 CC genotype of microRNA-146a is linked to a high risk of grade 3 or higher irAEs [202,208].

## 4. Conclusions and Discussion

The immune system is equipped with highly specific molecules and cells to combat pathogenic, genetic, and metabolic diseases. The innate and adaptive cascades are efficient in protecting against cancer antigens, yet cancer cells have evolved escape and resistance mechanisms in the complex TME [5]. The organization and homeostasis of cellular and non-cellular components of the TME enhance angiogenesis to support the transport of nutrients/oxygen, removal of metabolic waste, and attract pro- and anti-tumor immune cells to the neoplastic tissue. In the highly dynamic and complex TME, innate and adaptive immune cells, cytokines, chemokines, and pathophysiological conditions greatly influence tumor formation and progression. Therefore, a better understanding of immunotherapy is crucial for effective treatment strategies for a large variety of cancers [56,57,58].

Several treatment procedures for cancer, including surgery, radiation, cytotoxic chemotherapy, and immunotherapy, are deployed alone or in combination for improved outcomes and patient survival. Immunotherapy is relatively new and, in the last few decades, has evolved into different forms, and novel immunotherapy strategies are in the trial phase. The different cancer immunotherapies deployed/in the trial phase include cancer vaccines for activation of antitumor T cell responses, cytokine-induced antitumor responses, adoptive cell therapy, and ICI molecule-based therapy. CAR-engineered molecules with highly specific anti-cancer abilities have been incorporated into immune cells, which include T cells, NK cells, and macrophages. CAR-T-cell therapies have proved promising against hematological malignancies [21,25,27,214]. Despite continuous optimization of CAR T-cell therapies, their implementation is still limited in solid tumors [33]. Additionally, life-threatening toxicities like cytokine release syndrome (CRS) and neurotoxicity (ICANS), modest anti-tumor activity, antigen escape, limited tumor infiltration, and altered CAR-T cell function are limiting factors in the applicability of CAR-T cell therapies [215].

ICIs, improving antitumor immune responses by suppressing the coinhibitory signaling pathways, demonstrate a broad and stable efficacy in metastatic and chemotherapy-resistant tumors [216]. The inhibition of CTLA-4, PD-1, and PD-L1 is widely implemented in ICI therapy [41]. Resistance to ICIs and associated toxicities are the major obstacles in ICI cancer immunotherapies [53,54]. Several immunotherapies alone or in combination have proved highly promising, including solid and metastatic cancers, particularly for NSCLC.

NSCLC, the predominant type of lung cancer, has shown significant improvement in survival with immunotherapy over the past decade. Thus, immunotherapy is approved for metastatic cancer as the first-line single agent or in combination, in the adjuvant setting following chemotherapy and radiation as well as in the neoadjuvant setting for early-stage resectable tumors [121]. However, neoadjuvant immunotherapy proved more efficient, regardless of the patient’s PD-L1 status and histology subtype. The neoadjuvant effectiveness is potentially attributed to the large number of antigens present on the primary tumor, for T cell priming [125,128,132,217]. However, one of the major limiting factors in immunotherapy’s applicability is the large and diverse range of irAEs.

ICI therapies are associated with mild to life-threatening irAEs in the form of inflammatory and autoimmune reactions [167]. Major risk factors contributing to irAEs have been recognized in several cancer types, including not only pathogenicity but also pre-existing genotypes, mutations, inflammation and other health conditions in different organ systems. Although fatal irAEs are rare, pneumonitis, hepatitis, and colitis are the predominant irAEs responsible for mortality in the ICI treatment of NSCLC [179]. Definitive detection assays for specific irAEs predictions are not available, due to the unknown definitive mechanism of irAE development [169]. Currently, blood and serum analysis for autoantibodies and cytokines, as well as genetic mutation and HLA genotype assessments, provide a potential approach to assess the risk of developing irAEs [202].

Cancer immunotherapies are continuously evolving to combat unresolved challenges and hurdles for the evaluation of single/combo immunotherapies and the associated irAEs. The underlying factors responsible for varying response of the immunotherapies, notably variable antigens, dynamic TMEs, immune modulators, gut microbiota and co-pathogenicity, are yet to be explored further. Overall, the lack of predictable efficacy of immunotherapy, predictable response of the patient, efficient medical assessment plans, specific biomarker availability, evidence of profound improvement of immunity against malignancies, and high therapy costs are the major obstacles toward the broader applicability of cancer immunotherapies [218].

Eradication of immunosuppressive cells (TAMs and Tregs), efficient immune cell infiltration mechanisms, and modulation of the cytokine profiles and biochemical factors in the TME are potential targets for efficient immunotherapies. Furthermore, combining immunotherapies with traditional methods or harnessing the gut microbiome, personalized immunotherapies against neoantigens, selective targets through oncolytic viruses, and tailored cancer vaccines are emerging immunotherapeutic approaches for improved efficacy and overcoming resistance. Identification of new ICs, CAR-NK cell therapy, ICIs in combination with CAR-T cell therapy for enhanced and persistent efficacy in solid tumors, engineered CAR-T cells capable of self-sustained cytokine production, dual-targeting CAR-T cells against multiple antigens, and universal CAR-T cells are in the phase of exploration. CAR-NK does not require HLA compatibility, has relatively low safety concerns, and demonstrates promising results in hematological malignancies with limited efficacy in solid tumors [219]. Dual-target CAR-T poses a reduced risk of antigen escape by targeting two tumor antigens simultaneously, thus enhancing treatment efficacy and reducing the possibility of disease relapse [220]. Universal CAR-T cells, derived from healthy allogeneic donors, offer an immediate ready-to-use therapy option. However, graft-versus-host disease (GvHD) and host-versus-graft rejection (HvGR) are the key immunological challenges to overcome for clinical translation [221]. Additionally, highly specific and improved detection tools for the irAEs are crucial in accelerating the development and approval of new immunotherapies. These future explorations have the potential to broaden the horizon of immunotherapy to a larger patient population, for durable and long-lasting cancer eradication [222,223].

## Figures and Tables

**Table 1:** Clinical trials of immune checkpoint inhibitors in NSCLC.

Clinical Settings	Clinical Trials	Cancer Stage/Type	Treatment	Outcome
Early-stage resected NSCLC (adjuvant therapy)	IMpower010	Resected stage II-IIIA NSCLC and PD-L1 > 1%	Atezolizumab vs. best supportive care	Improved disease-free survival (31.2-month difference)
	Keynote-091	Resected stage IB, II, or IIIA NSCLC regardless of PD-L1 status	Pembrolizumab vs. placebo	Improved disease-free survival (53.6 vs. 42 months)
Neoadjuvant/Perioperative immunotherapy	Checkmate 816 (Neoadjuvant only)	Resectable stage IB (≥4 cm)-IIIA NSCLC	Nivolumab and chemotherapy vs. chemotherapy	Improved median event-free survival (31.6 vs. 20.8 months) and higher Rates of pathological complete responses (pCR 24.0% vs. 2.2%)
	Checkmate 77T	Resectable stage IIA-IIIB NSCLC	Perioperative nivolumab plus chemotherapy vs. chemotherapy	Event-free survival benefit (18-month EFS 70.2% vs. 50.0%)
	NEOSTAR	Resectable stage IA-IIIA NSCLC	Ipilimumab and nivolumab combination vs. nivolumab	Increased major pathological response (MPR 38% vs. 22%)
	NADIM II	Resectable stages IIIA and IIIB NSCLC	Perioperative platinum doublet with nivolumab vs. chemotherapy	Higher rates of complete pathological responses (37% vs. 7%) and PFS (24-month PFS was 67.2% vs. 40.9%; HR 0.47) and improved OS at 24 months (85.0% vs. 63.6%; HR 0.43)
	Keynote 671	Resectable stages II, IIIA, and IIIB NSCLC	Perioperative pembrolizumab and cisplatin doublets vs. chemotherapy	Significant improvement in event-free survival, major and complete pathological response and overall survival
	Neotorch	Resectable stage III NSCLC	Perioperative toripalimab plus chemotherapy vs. placebo plus chemotherapy	Significant improvement in event-free survival
Locally advanced or metastatic NSCLC	PACIFIC	Unresected Stage III NSCLC	Chemo-radiotherapy followed by Durvalumab vs. placebo	Significantly improved PFS (16.9 vs. 5.6 months) and median OS (47.5 vs. 29.1 months)
	Keynote 024	NSCLC with TPS ≥ 50%	Pembrolizumab vs. platinum doublet	Median OS improved (26.3 months vs. 13.4 months)
	IMpower 110	NSCLC with TC ≥ 50%	Atezolizumab vs. chemotherapy	Improved median OS (20.2 months vs. 13.1 months)
	EMPOWER-Lung-1	Unresected stage IIIB, IIIC and IV NSCLC with PD-1 and TPS ≥ 50%	Cemiplimab vs. chemotherapy	Improved median OS (26.1 months vs. 13.3 months)
	Keynote 189	Metastatic non-squamous NSCLC without sensitizing EGFR and ALK mutations	Pembrolizumab and platinum-pemetrexed vs. placebo and platinum pemetrexed	Increased response rates and better OS (at 5 years 19.4% vs. 11.3%)
	Keynote 407	Squamous NSCLC	Pembrolizumab with chemotherapy vs. placebo and chemotherapy	Improved PFS (6.4 months vs. 4.8 months), and increased median OS (15.9 months vs. 11.3 months)
	IMpower 130	Stage IV non-squamous NSCLC without EGFR and ALK mutations	Atezolizumab and chemotherapy vs. chemotherapy	Improved PFS (7.0 months vs. 5.5 months) and median OS (19.5 months vs. 14.7 months)
	IMpower 150	Metastatic NSCLC	Carboplatin doublet with atezolizumab and bevacizumab vs. doublet with bevacizumab	Improved median OS (19.5 months vs. 14.7 months)
	Checkmate 227	Advanced NSCLC	Ipilimumab and nivolumab vs. chemotherapy	Improved median OS (17.1 months vs. 13.9 months)
	Checkmate 9LA	Stage IV or recurrent NSCLC	Chemotherapy with ipilimumab and nivolumab vs. chemotherapy	Improved median OS (15.6 months vs. 10.9 months)
	Phase III POSEIDON	NSCLC with mutations like STK11 and KEAP1	Durvalumab, tremelimumab, and chemo vs. chemo	Improved PFS and OS (PFS 6.2 vs. 4.8 months and OS 14.0 vs. 11.7 months)
	MYSTIC	Metastatic NSCLC without sensitizing EGFR and ALK mutations	Durvalumab and tremelimumab vs. chemo	No improvement of outcomes
Radiation therapy and ICI	Phase-II PEMBRO-RT and MDACC pooled analysis	Metastatic NSCLC	Pembrolizumab and radiotherapy vs. pembrolizumab alone	Improved overall response rate (36% vs. 18%)
CNS disease and ICI	Atezo-Brain	Non-squamous NSCLC with untreated brain metastases	Atezolizumab plus carboplatin and pemetrexed	Median OS of 11.8 months

## Data Availability

No new data were created or analyzed in this study. Data sharing is not applicable to this article.
